# Exercise Perceptions, Barriers, and Self-Efficacy Among Adults in Kuwait During the COVID-19 Pandemic

**DOI:** 10.3390/ijerph23040462

**Published:** 2026-04-04

**Authors:** Naser A. Albazzaz, Abdulaziz Alhenaidi, Mohammad Almari, Ahmad Aldahas, Sultan E. Alsalahi

**Affiliations:** 1 Quality and Medical Cooperation Department, Medical Services Authority, Ministry of Defense, Kuwait City P.O. Box 100010, Kuwait; nasseral-bazzaz@hotmail.com; 2 Ministry of Health, Kuwait City P.O. Box 13001, Kuwait; aalhenaidi@moh.gov.kw; 3 Department of Health Policy & Management, College of Public Health, Kuwait University, Kuwait City P.O. Box 13110, Kuwait; mohammad.almari@ku.edu.kw; 4 Department of Physical Therapy, Alsabah Medical Hospital, Ministry of Health, Kuwait City P.O. Box 13001, Kuwait; aldahas9916@moh.gov.kw; 5 Department of Physical Therapy, Alrazi National Orthopaedic Hospital, Ministry of Health, Jamal Abdulnasser Street, Kuwait City P.O. Box 13001, Kuwait

**Keywords:** physical activity, behavioural psychology, public health, COVID-19 lockdown

## Abstract

**Highlights:**

**Public health relevance—How does this study relate to a public-health issue?**

- This study focuses on the psychosocial factors associated with exercise participation among adults in Kuwait during the COVID-19 pandemic, a period during which strict public-health restrictions limited opportunities for physical movement and increased physical inactivity.
- This study identifies the relationship between benefits, barriers, and self-efficacy among exercisers and non-exercisers among adults in Kuwait during the COVID-19 pandemic.

**Public health significance—Why is this study of significance to public health?**

- This study demonstrates that higher percieved exercise benefits, barriers and barrier-specific self-efficacy were significantly associated with exercising among Kuwaiti adults during the COVID-19 pandemic.
- This study highlights that adults with higher education, who were employed, or who were single were more likely to participate in exercise during the COVID-19 pandemic.

**Public-health implications—What are the key implications or messages for practitioners, policy makers, and/or researchers in public health?**

- Public-health strategies during viral pandemic should focus on enhancing awareness around psychological and physical benefits of exercise and improving self-efficacy.
- Policy makers in Kuwait should concentrate public-health messages around cognitive-behavioural interventions and address socioeconomic and contextual barriers to enhance exercise participation.

**Abstract:**

The COVID-19 pandemic led to strict public-health restrictions that affected opportunities for physical movement, including exercise participation. Understanding psychosocial factors of exercise under these conditions is vital for developing exercise promotion strategies. This study aimed to compare exercise perceptions and self-efficacy between exercising and non-exercising adults (>21 years old) in Kuwait during the COVID-19 pandemic. A cross-sectional online survey was conducted among adults living in Kuwait during June and July 2020. Exercise status was self-reported based on a 150-min weekly exercise threshold. Participants completed the Exercise Benefits/Barriers Scale (EBBS) and the Barriers-Specific Self-Efficacy Scale (BARSE). Data were analysed using independent *t*-tests to assess group differences and multivariable binary logistic regression to identify factors independently associated with being an exerciser. A total of 929 individuals participated in the study, of which 54% were categorised as exercisers. Exercisers reported significantly higher perceived benefits, barriers, and BARSE scores compared to non-exercisers. Binary logistic regression indicated that higher perceived benefits and barriers and higher BARSE scores were significantly associated with exercise status. In addition, holding a diploma or university education, postgraduate education, being employed, and being single were associated with higher odds of engaging in exercise while older adults were associated with lower odds of engaging in exercise. Positive exercise perceptions and higher barrier-specific self-efficacy were significantly associated with exercise participation during the COVID-19 pandemic in Kuwait.

## 1. Introduction

Noncommunicable diseases (NCDs) are one of the leading causes of mortality and morbidity in Kuwait [1]. Physical inactivity is considered one of the main modifiable risk factors for NCDs such as cardiovascular disease, type 2 diabetes, and obesity [2,3,4,5,6]. Kuwait has one of the highest global prevalence rates of physical inactivity, with a report demonstrating 67% of adults in Kuwait not engaging in weekly physical activity [7,8].

During the COVID-19 pandemic, unprecedented restrictions were implemented to mitigate transmission and relieve the pressure on health services, leading to substantial changes in daily life [9,10]. Measures such as lockdowns, curfews, closure of gyms, and outdoor restrictions impacted movements and limited social interaction, leading to reduced physical activity levels and increased incidences of sedentary behaviour [11,12]. For example, a longitudinal pedometer study in a neighbouring country, Qatar, reported reductions of up to 1300 steps per day during the COVID-19 lockdown [13]. Furthermore, a cross-sectional study using the International Physical Activity Questionnaire conducted in Kuwait during COVID-19 restrictions found low physical activity levels among adults in Kuwait [14]. Given these changes in physical activity levels, it is important to identify factors that can influence exercise engagement in these restricted conditions. While demographic and environmental factors influence behaviour, cognitive-perceptual factors are modifiable predictors of behaviour change [15]. Therefore, to understand these changes, it is crucial to investigate psychological mechanisms that influence exercise participation under such constrained conditions.

Psychological factors such as perceived benefits, barriers, and self-efficacy are key drivers of individual exercise behaviours [16,17]. Perceived benefits are defined by how an individual perceives the positive outcomes of exercise, while barriers include both physical and psychological obstacles that impede exercise initiation. The Health Belief Model (HBM) suggests that perceived benefits outweighing barriers is key to initiating exercise [18,19]. These perceptions are directly influenced by modifying factors such as age, education, and employment. Previous research using the EBBS scale has shown that higher physical activity is associated with higher perceived benefits and lower perceived barriers to exercise among adult and adults [20,21].

On the other hand, Social Cognitive Theory (SCT) emphasises self-efficacy, which is defined as an individual confidence to perform an action despite barriers and is a primary determinant of action in the face of barriers. Indeed, a meta-analysis confirmed that self-efficacy interventions increased physical activity engagement [22]. Furthermore, higher self-efficacy to overcome barriers to exercise measured using the BARSE scale showed better exercise engagement [23,24]. However, no study in Kuwait has identified psychological factors of exercise during the pandemic. Therefore, understanding exercise perceptions, identifying barriers to physical activity, and evaluating self-efficacy are essential for promoting engagement in regular exercise among adults in Kuwait, particularly in the context of pandemic-related restrictions.

Understanding exercise perceptions, identifying barriers to physical activity, and evaluating self-efficacy during a pandemic have important implications for Kuwait’s national health strategy and its position within the WHO Global Action Plan on Physical Activity 2018–2030 [25]. Several studies have documented a range of barriers to physical activity, including family responsibilities and laziness, among both adults and adolescents [26,27,28]. However, no studies have investigated psychosocial predictors of exercise during the pandemic in Kuwait. One local survey has found that better mental status measured using the WHO-5 wellbeing index was associated with higher levels of physical activity [29]. However, this study did not assess exercise perceptions, barriers to physical activity, or self-efficacy. Evidence on these factors is needed and can help inform national physical activity initiatives to align them with WHO recommendations.

Despite reports of physical inactivity in Kuwait during the pandemic, the psychosocial predictors of exercise engagement remain unclear. No study has previously investigated psychosocial factors that influence exercise during the pandemic. Furthermore, comparisons between exercisers and non-exercisers can detect psychological gaps in perception and self-efficacy. In Kuwait, during the first wave of viral infections in 2020, the government implemented strict lockdowns and a 24-h curfew during which all non-essential businesses were closed and a strong stay-at-home message was enforced [30]. However, during June and July of the same year, the curfew was reduced to a 12 h [31]. Therefore, grounded in the theoretical frameworks of the HBM and SCT, the objective of this study was to compare exercise perceptions, barriers, and self-efficacy among adults who exercised and those who did not engage in exercise during the COVID-19 pandemic in Kuwait. Understanding these factors may help guide public-health strategies to address inactivity during any future pandemics.

## 2. Materials and Methods

This cross-sectional study employed an online survey administered via Google Forms distributed via social media platforms (X and WhatsApp) during June and July of 2020, using a sample of convenience. An electronic link was accessed by participants, to which they responded on the same day, including an electronic informed consent before starting the survey. Informed consent was obtained from all subjects involved in the study. The survey website allowed one response per user and multiple submissions were restricted. Sociodemographic and health-related data were self-reported, including gender, age, height and weight, marital status, whether participants engage in 150 min of exercise per week, and smoking. In addition, the residential governorate was recorded, representing the official administrative governate that covers the entire State of Kuwait. Inclusion criteria involved any participants living in the state of Kuwait and aged 21 years or older. Participants with mobility impairment or a condition that had restricted their daily activity and movement in the last 7 days were excluded from the study.

### 2.1. Participant Characteristics

Table 1 and Table 2 present the distribution of participants’ sociodemographic and clinical characteristics according to exercise status. A total of 1019 participants took part in the study, and 90 were excluded due to incomplete responses or not meeting the inclusion criteria, resulting in 929 participants included for the analysis. The majority of participants were female (57%), while 43% were male. Most of the participants were in the age group of 21–35 years old (60%), while age groups of over 35 to 45 years old and over 45 years of age were 17% and 24%, respectively. Regarding education, most participants held either a diploma or a university degree (77%), while 17% were postgraduates, and 7% had completed high school or less. Regarding exercise status, 428 (46%) of the participants were considered non-exercisers, while 497 (54%) were exercisers. Most participants resided in either Kuwait City (25%), Hawally (23%), or Mubarak Alkabeer (21%), while the others lived in Farwaniya (13%), Alahmadi (12%), and Aljahra (7.5%). With respect to social status, 54% were married, 42% were single, and 5% reported either being divorced or widowed. The majority of participants had no children (48%), while 18% reported having one or two children, 20% having three or four, and 14% reported having more than five children. In terms of employment status, 67% of participants were employees, 19% were students, 8% were retired, and 6% reported other employment statuses. BMI results show that most participants were either obese (27%) or overweight (38%), while 35% had a normal BMI or were underweight. Most participants were non-smokers (78%), while 22% were smokers.

### 2.2. Sample Size Calculations

Based on a target population of 3,706,000 [32] and assuming 95% confidence intervals with a 5% margin of error, a minimum of 384 participants was required.

### 2.3. Ethical Approval

The study was conducted in accordance with the Declaration of Helsinki and approved by the Ethical and Health Ethical Committee of the Ministry of Health of Kuwait (2020/1501).

### 2.4. Instruments

Since EBBS and BARSE were originally developed in English (See Tables S1 and S3, Supplementary Materials), a forward-backward translation into Arabic was employed as per [33]. Two bilingual health professionals translated both surveys into Arabic, and an independent translator translated both Arabic versions back into English to ensure consistency. Internal consistency of the Arabic versions of EBBS (See Table S2, Supplementary Materials) and BARSE (See Table S4, Supplementary Materials) scales were evaluated using Cronbach’s alpha. The 43-item EBBS total scale demonstrated excellent internal consistency (α = 0.93), while the 13-item BARSE scale showed good internal consistency (α = 0.89). This confirms that both scales translation were valid for the population in Kuwait.

EBBS and BARSE are widely used instruments that assess exercise benefits, barriers, and confidence in engaging in exercise when facing common barriers to exercise [34,35,36]. The EBBS is a 43-item scale to assess perceived barriers and benefits to exercise, including 29 benefits items, and 14 barrier items using a 4-point Likert scale ranging from 1 (strongly disagree) to 4 (strongly agree). Barriers items are reversed-scored from 4 (strongly disagree) to 1 (strongly agree), and high scores indicated a more positive perception of exercise. The range of scores is from 43 to 172, with higher scores indicating favourable exercise perceptions [33]. EBBS has been widely used to assess exercise perceptions [37] and has demonstrated good internal consistency and construct validity in adults [38] and older populations [36]. Self-efficacy of exercise was measured using the BARSE scale, which assesses confidence in exercising three times per week for 40 min over the next 2 months despite facing common barriers (e.g., bad weather, lack of time, lack of facilities). Participants rate their confidence on a 100-point percentage scale comprising 10-point increments from 0% (not at all confident) to 100% (highly confident). Items are summed up and a higher total score indicates greater confidence in exercising. The BARSE scale has been widely used to assess exercise self-efficacy [39] and has demonstrated good internal consistency [40] and construct validity in adults and older populations [35,41].

Participants’ exercise status was determined by a single question: “Do you usually perform at least 150 min per week of exercise?”, with a response option of Yes/No. Participants answering no were considered as non-exercisers and participants answering yes were considered as exercisers. This single-item question has been used previously to provide a simple classification approach to distinguish between exercisers and non-exercisers [42]. Furthermore, the cut-off of 150 min per week of exercise chosen in this study is an attempt to approximate the minimum weekly volume of physical activity required as per WHO recommendations to achieve health benefits [43].

### 2.5. Data Analysis

Data were inspected, cleaned, and analysed, and missing data or incomplete responses were removed. Continuous variables were computed as means and standard deviations (SD) and categorical variables as frequencies and percentages. Normality was assessed using visual inspections of histograms. Group differences in EBBS and BARSE between exercisers and non-exercisers were analysed using independent-samples *t*-tests. Multivariable analysis used binary logistic regression with exerciser status as the dependent variable. Predictors included were EBBS, BARSE, gender, governorate, social status, number of children, employment status, smoking status, education level, age group, and body mass index (BMI) group. The addition of sociodemographic factors (e.g., age, education) was based on HBM, which hypothesise them as ‘modifying factors’ that influence health perceptions. On the other hand, psychological variables (EBBS and BARSE) were involved as direct behavioural determinants consistent with SCT. Age was categorised into three groups (21–35, 36–45, and >45 years) to better reflect differences between early adulthood, peak family or career responsibility, and the pre-retirement period. Educational levels were categorised into three groups to attain the well-established differences in health literacy [44]. The following groups were used as reference categories: male (gender); 21–35 years (age group); high school or less (education); Kuwait City (governorate); married (social status); no children (number of children); student (employment status); and <25 kg/m^2^ for BMI. BMI was calculated using the following formula after converting height to metres: BMI = kg/m^2^, and the participants were classified into categories (<25, 25–30, >30). Odds ratios (ORs) with 95% confidence intervals (CIs) were reported. All analyses were performed using the Statistical Package for the Social Sciences (SPSS IBM version 30.0, IBM Corporation, Armonk, NY, USA). Results were considered statistically significant when *p* < 0.05.

## 3. Results

### 3.1. Exercise Perceptions/Self-Efficacy

The mean EBBS perceived benefits for the exercisers group was 98.7 ± 11.7, while the mean for non-exercisers group was 90.2 ± 12.2. The independent sample *t*-test revealing the difference in means between both groups was significant (*p* < 0.001; Cohen’s d = 0.71; *t* = 10.8). The mean EBBS perceived barriers score for the exercisers group was 42.5 ± 6.5, while the mean for non-exercisers group was 38.0 ± 6.4. The independent sample *t*-test revealing the difference in means between both groups was significant (*p* < 0.001; Cohen’s d = 0.70; *t* = 10.7). The mean BARSE for exercisers was 76.2 ± 26.1, while BARSE for non-exercisers was 61.3 ± 27.8. The independent sample *t*-test revealed significant differences between both groups (*p* < 0.001; Cohen’s d = 0.55; *t* = −8.4).

### 3.2. Regression Analysis

**Figure 1:**
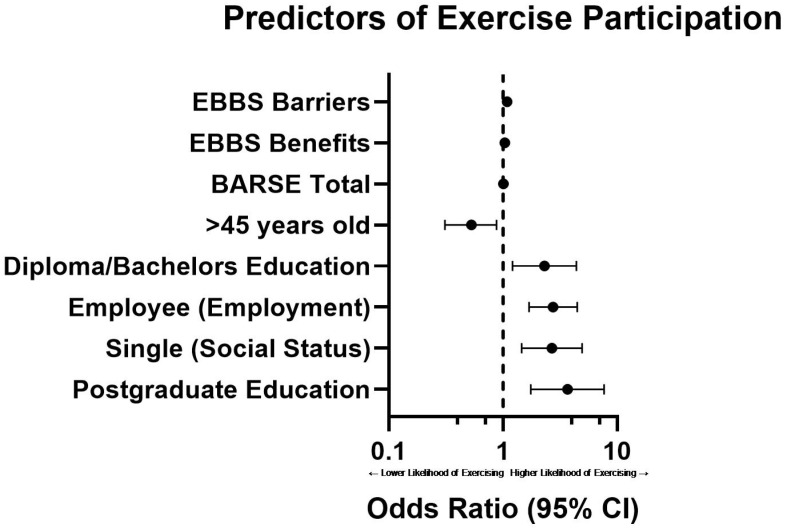
Forest plot showing adjusted odds ratios (ORs) and 95% confidence intervals from a multivariable binary logistic regression model with exercise participation as the dependent variable. EBBS Total and BARSE Total are continuous scores for perceived exercise benefits and exercise self-efficacy, respectively. Diploma/university education and postgraduate education are compared with high school or less as the reference category. Employee (employment) is compared with student as the reference employment category. The >45 years age group is compared with the youngest group. Single (social status) is compared with married as the reference social status. All ORs are adjusted for governorate, number of children, BMI group, age group, smoking status, sex, and the other variables in the model.

The multivariable binary logistic regression model results are depicted in Figure 1. Results revealed that higher scores on EBBS subscales and BARSE scores were significantly associated with greater odds of being an exerciser. Specifically, one point increase in perceived benefits increased the odds of being an exerciser by 4% (*p* < 0.001; OR = 1.04, 95% CI 1.02–1.05), while one point increase in perceived barriers increased the odds of by 9% (*p* < 0.001; OR = 1.09, 95% CI 1.06–1.12) and one point increase in BARSE increased the odds of being an exerciser by 1.4% (*p* < 0.001; OR = 1.01, 95% CI 1.00–1.02). Participants with diploma or university education (*p* = 0.011; OR = 2.31, 95% CI 1.21–4.40) and postgraduate education were more likely to be an exerciser compared to participants with a high-school education or less (*p* < 0.001; OR = 3.68, 95% CI 1.75–7.70). Furthermore, participants that are employed showed higher odds for engaging in exercise compared to students (*p* < 0.001; OR = 2.75, 95% CI 1.69–4.47). Single adults had significantly higher odds of being an exerciser compared to married participants (*p* = 0.001; OR = 2.69, 95% CI 1.46–4.94), while all other categories in social status were not significant compared to married participants. Also, age was significantly associated with exercise status, where the oldest group (>45 years old) showed significantly lower odds of exercising compared to the youngest group (*p* = 0.033; OR = 0.53, 95% CI 0.31–0.88). All other demographic and health variables were not statistically associated with exercise after adjustment for all variables.

## 4. Discussion

This is the first study to investigate exercise perceptions, barriers, and self-efficacy and identify factors associated with exercising between exercisers and non-exerciser among adults in Kuwait during the COVID-19 pandemic. The main findings revealed that during the COVID-19 pandemic, higher self-efficacy and a more positive exercise perception were significantly associated with engagement in exercise, in addition to higher education levels, being single, and being employed.

The results of this study are consistent with health behaviour theoretical models that associate perceived benefits barriers and self-efficacy with engagement in exercise [16,18,19,45,46]. The results of the binary logistic regression have revealed that a one-point increase in perceived benefits measured by EBBS and self-efficacy measured by BARSE increased the odds of being an exerciser by 4% and 1%, respectively. However, the EBBS subscale analysis showed a nuanced behavioural profile. While the exercising group revealed a higher perception of the benefits of exercise, they also reported higher perceived barriers compared to the non-exercising group. Individuals who perceive the psychological and physical benefits of exercise and recognise that barriers to exercise can be managed, as supported by higher benefits and higher perception of barriers of exercise and BARSE scores, are more likely to participate in exercise. On the other hand, individuals that do not perceive those benefits to exercise are less likely to engage in physical activity and perform exercise. Notably, perception of exercise measured by EBBS showed larger effect sizes between groups (Cohen’s d = 0.71 for benefits, d = 0.70 for barriers) than self-efficacy (d = 0.55), indicating that, during lockdown, individuals’ evaluation of benefits versus barriers was a more prominent discriminator of exercising behaviour than self-efficacy. The results of the EBBS scores highlight a perceptual gap between exercisers and non-exercisers during the pandemic lockdown.

All participants experienced similar environmental conditions, including extreme heat conditions in Kuwait, combined with lockdowns. This presented a double barrier to exercise engagement as participants had to cope with restrictions and extreme weather conditions. Nevertheless, exercisers perceived substantial benefits of exercise and viewed barriers as manageable, indicating the cognitive resilience is associated with engagement in exercise during these conditions. Regional studies indicated that extreme high temperature is identified as a barrier to exercise engagement in Arab countries [47,48]. Given the high perception of benefits of exercise, exercisers have appeared to adopt compensatory strategies to engage in exercise. Although this study did not investigate how participants were engaging in exercise during these barriers, it is possible that exercisers may have adopted home-based exercise regimens or engaged in exercise during the evening hours when temperatures were cooler.

Another key finding of this study was that education and employment were found to be significantly associated with exercise participation. Participants with a diploma or university degree (OR = 2.31; 95% CI 1.21–4.40), as well as those with postgraduate education, were nearly four times more likely to engage in exercise than participants with a high-school education or less (OR = 3.68; 95% CI 1.75–7.70). This finding suggests that higher education may be associated with higher health literacy and awareness of the benefits of exercise. Previous studies have reported that higher education is linked to a higher likelihood of meeting the physical activity recommendations and is associated with a lower likelihood of being physically inactive [49,50,51]. In our study, higher education was significantly associated with being categorised in the exercise group (≥150 min per week).

Furthermore, employed participants were more likely to engage in exercise than students, those who were unemployed, or retirees (OR = 2.75; 95% CI 1.69–4.47). This agrees with other studies that have reported that being unemployed and being a student were associated with a higher risk of physical inactivity [20,47,49,51,52,53,54]. Possible explanations include poorer mental health and reduced motivation among the unemployed or study-related stress among students, which may act as barriers to physical activity engagement [55,56]. In this sample, the complex interplay between employment and education status suggests that socioeconomic and psychological barriers warrant further investigation.

An interesting finding of this study is that gender and BMI were not significantly associated with exercise status. However, age was confirmed as a significant factor, with older adults demonstrating lower odds of exercising compared to younger adults (*p* = 0.001; OR = 2.69, 95% CI 1.46–4.94). This aligns with previous pre-pandemic regional studies which reported that older adults are at higher risk of physical inactivity [57,58,59]. The lack of significance across gender may suggest that restrictions may have reduced gender specific barriers, reinforcing the notion that self-efficacy is associated with exercise engagement regardless of gender or age. One possible explanation is that during restrictions, traditional barriers that would affect gender, such as few segregated outdoor environments or the promotion of online home-based programmes, may have reduced the differences between age and gender. Indeed, during the same period, the use of online home-based programme applications in increased in Kuwait and reports described it as the new norm in Kuwait and other GCC countries [60].

### 4.1. Policy Recommendations

The findings of this study have several practical and policy implications. First, public-health messaging might enhance exercise engagement by focusing on the immediate psychological benefits of exercise and reframing exercise as a psychological and wellbeing intervention rather than just a weight-management tool.

Second, because students were considered in the high-risk demographic, universities could integrate health promotion messaging across campuses by rewarding exercise participations and encouraging the use of wellness and gym facilities on campuses. Third, on a national level, exercise participation messaging could generate strategies that address heat as a barrier to exercise participation by offering alternatives that can increase participation, such as climate-controlled or other weather-protected venues for exercise.

### 4.2. Limitations

This study has several limitations. First, the cross-sectional design of the study and the time-point at which it was conducted, during the first wave of restrictions, limit causal inferences between the variables studied and self-efficacy or exercise determinants or the generalisabilty of results to the whole pandemic period. However, the data offer a valuable historical baseline for understanding exercise participation factors during acute pandemic restrictions rather than identifying long term behavioural patterns. The classification of exercisers from non-exercisers was determined using a single self-reported question regarding participants engaging in 150 min of total weekly exercise. This item does not specify the intensity of exercise according to the WHO physical activity guidelines, which may under- or over-estimate the ratio of participants meeting WHO weekly recommendations. However, such misclassification is a common limitation concerned with all self-reported measurements when compared to gold-standard measurements such as accelerometry [61]. During this difficult pandemic period, infection control considerations made it logistically impossible to plan an accelerometry study. Furthermore, self-reported questionnaires regarding physical activity levels have shown acceptable agreement when compared with direct measures such as an accelerometer [62]. Another limitation associated with self-reported questionnaires is the risk of information bias and social desirability bias; participants may alter their reported perception of exercise to align with social expectations. However, the use of an anonymous online survey likely attenuated this tendency compared to face-to-face administration [63]. The other limitation concerned with the study is the sampling method, which might introduce sampling bias favouring a demographic of a younger, more digitally literate, and more educated population. However, due to stay-at-home messaging and avoiding non-essential contact, the use of this type of sampling was a feasible strategy. In this study, the main purpose of this item was to provide a distinction between exercisers and non-exercisers rather than quantify prevalence. Hence, it showed good discriminatory ability where patterns of exercisers were consistent with established links with regards to higher perceived benefits and awareness of barriers, compared to the non-exercising group [64].

## 5. Conclusions

In conclusion, during the COVID-19 pandemic in Kuwait, exercisers reported higher perceived benefits and barriers to exercise relying on stronger self-efficacy to engage in exercise. The findings of this study also showed that adults with a higher level of education, those who were employed, and those who were younger in age or single were more likely to engage in exercise. Public-health strategies during a viral pandemic should focus on reframing exercise around psychological and physical wellbeing, and targeting high-risk demographics such as students, married individuals, and unemployed individuals, in addition to addressing environmental barriers to enhance exercise participation.:

## Figures and Tables

**Table 1:** Individual characteristics by exercise status. Distribution of participants’ sociodemographic characteristics according to exercise status (Yes/No). Exercise status was defined as meeting ≥150 min per week of exercise. Values are presented as the number and percentage of participants within each category of gender, age group, education level, employment status, and social status.

		Exercise Status		Total
		No	Yes	
**Gender**	**Male**	168	233	401
		39%	47%	43%
	**Female**	262	266	528
		61%	53%	57%
**Age Group**	**21–35 years old**	224	328	552
		52%	66%	59%
	**35–45 years old**	84	74	158
		20%	15%	17%
	**over 45 years old**	122	97	219
		28%	19%	24%
**Education**	**High-school or Less**	45	18	63
		10%	4%	7%
	**Diploma or Bachelors**	326	386	712
		76%	77%	77%
	**Postgraduate**	59	95	154
		14%	19%	17%
**Social Status**	**Married**	270	227	497
		63%	45%	53%
	**Single**	143	244	387
		33%	49%	42%
	**Other**	17	28	45
		4%	6%	5%
**Employment**	**Student**	88	87	175
		20%	17%	19%
	**Employee**	270	356	626
		63%	71%	67%
	**Retired**	41	28	69
		10%	6%	7%
	**Other**	31	28	59
		7%	6%	6%

**Table 2:** Household and contextual characteristics by exercise status. Distribution of household- and context-related characteristics according to exercise status (Yes/No). Exercise status was defined as meeting ≥150 min per week of exercise. Values are presented as the number and percentage of participants within each category of gov.

		Exercise Status		Total
		No	Yes	
**Number of Children**	**No Children**	175	275	450
		41%	55%	48%
	**1 or 2**	82	85	167
		19%	17%	18%
	**3 or 4**	96	87	183
		22%	17%	20%
	**>5**	77	52	129
		18%	10%	14%
**Governorate**	**Kuwait City**	98	135	233
		23%	27%	25%
	**Alahmadi**	56	51	107
		13%	10%	12%
	**Farwaniya**	60	59	119
		14%	12%	13%
	**Aljahra**	35	34	69
		8%	7%	7%
	**Hawaly**	105	104	209
		24%	21%	22%
	**Mubarak Alkabeer**	76	116	192
		18%	23%	21%
**BMI**	**<25**	157	167	324
		37%	34%	35%
	**25–30**	162	186	348
		38%	37%	38%
	**>30**	109	144	253
		25%	29%	27%
**Smoking**	**No**	333	388	721
		77%	78%	78%
	**Yes**	97	111	208
		23%	22%	22%

## Data Availability

The raw data supporting the conclusions of this article will be made available by the corresponding author on request.

## Supplementary Materials

The following supporting information can be downloaded at: https://www.mdpi.com/article/10.3390/ijerph23040462/s1, Table S1: The Exercise Benefits and Barriers Questionnaire (English Version); Table S2: The Exercise Benefits and Barriers Questionnaire (Arabic Version); Table S3: Barriers Specific Self-Efficacy Scale (BARSE) (English Version); Table S4: Barriers Specific Self-Efficacy Scale (BARSE) (Arabic Version).

## Abbreviations

The following abbreviations are used in this manuscript:

COVID-19	Coronavirus disease 2019
DOAJ	Exercise Benefits/Barriers Scale
TLA	Barriers Self-Efficacy Scale
SPSS	Statistical Package for the Social Sciences (IBM SPSS Statistics)
NCDs	Noncommunicable disease(s)
HBM	Health Belief Model
SCT	Social Cognitive Theory
