# Supplementary material for: Exercise Perceptions, Barriers, and Self-Efficacy Among Adults in Kuwait During the COVID-19 Pandemic
Source: Int J Environ Res Public Health. 2026 Apr 4;23(4):462. doi: 10.3390/ijerph23040462 (PMC13116150; doi:10.3390/ijerph23040462)
Supplement: Supplementary file 1 [file ijerph-23-00462-s001.zip › S1.pdf]

Supplementary Material - Tables S1 and S2 present the 43-item questionnaire used to assess participants' perceived benefits of and barriers to physical activity. Respondents rate their level of agreement with each statement using a 4-point Likert scale: Strongly Agree (SA), Agree (A), Disagree (D), and Strongly Disagree (SD). The instrument is divided into two primary constructs: Benefits: Items focusing on the positive outcomes of physical activity, including physiological improvements (e.g., increased stamina, muscle strength, cardiovascular function), psychological well-being (e.g., decreased stress, improved mental health, relaxation), and social advantages. Barriers: Items identifying obstacles to physical activity, such as time constraints, physical exertion/fatigue, financial costs, environmental limitations, and lack of social support.

Table S1 - The Exercise Benefits and Barriers Questionnaire (English Version)

1. I enjoy exercise	SA A D SD
2. Exercise decreases feelings of stress and tension for me	SA A D SD
3. Exercise improves my mental health	SA A D SD
4. Exercising takes too much of my time	SA A D SD
5. I will prevent heart attacks by exercising.	SA A D SD
6. Exercise tires me.	SA A D SD
7. Exercise increases my muscle strength.	SA A D SD
8. Exercise gives me a sense of personal accomplishment.	SA A D SD
9. Places for me to exercise are too far away.	SA A D SD
10. Exercising makes me feel relaxed.	SA A D SD
11. Exercising lets me have contact with friends and persons I enjoy	SA A D SD
12. I am too embarrassed to exercise.	SA A D SD
13. Exercising will keep me from having high blood pressure.	SA A D SD
14. It costs too much to exercise.	SA A D SD
15. Exercising increases my level of physical fitness.	SA A D SD

16. Exercise facilities do not have convenient schedules for me.	SA A D SD
17. My muscle tone is improved with exercise.	SA A D SD
18. Exercising improves functioning of my cardiovascular system.	SA A D SD
19. I am fatigued by exercise.	SA A D SD
20. I have improved feelings of well being from exercise.	SA A D SD
21. My spouse (or significant other) does not encourage exercising.	SA A D SD
22. Exercise increases my stamina.	SA A D SD
23. Exercise improves my flexibility.	SA A D SD
24. Exercise takes too much time from family relationships.	SA A D SD
25. My disposition is improved with exercise.	SA A D SD
26. Exercising helps me sleep better at night.	SA A D SD
27. I will live longer if I exercise.	SA A D SD
28. I think people in exercise clothes look funny.	SA A D SD
29. Exercise helps me decrease fatigue.	SA A D SD
30. Exercising is a good way for me to meet new people.	SA A D SD
31. My physical endurance is improved by exercising.	SA A D SD
32. Exercising improves my self-concept.	SA A D SD
33. My family members do not encourage me to exercise.	SA A D SD
34. Exercising increases my mental alertness.	SA A D SD
35. Exercise allows me to carry out normal activities without becoming tired.	SA A D SD

36. Exercise improves the quality of my work.	SA A D SD
37. Exercise takes too much time from my family responsibilities.	SA A D SD
38. Exercise is good entertainment for me.	SA A D SD
39. Exercising increases my acceptance by others.	SA A D SD
40. Exercise is hard work for me.	SA A D SD
41. Exercise improves overall body functioning for me.	SA A D SD
42. There are too few places for me to exercise.	SA A D SD
43. Exercise improves the way my body looks.	SA A D SD

Table S2 - The Exercise Benefits and Barriers Questionnaire (Arabic Version)

يرجى وضع علامة على اختيار واحد من التالي (م ش = موافق بشدة ، م = موافق ، غ = غير موافق ، غ ش = غير موافق بشدة)

1. أنا أستمتع بممارسة التمارين الرياضية
2. التمارين تقلل من مشاعر الإجهاد والتوتر بالنسبة لي
3. التمارين تحسن صحتي العقلية
4. التمارين تأخذ الكثير من وقتي
5. سوف أحمي نفسي من النوبات القلبية عن طريق التمارين
6. التمارين تجهدني
7. التمارين تزيد من قوة عضلاتي
8. التمارين تعطيني شعوراً بالإنجاز الشخصي
9. أماكن التدريب بعيدة جداً عني
10.التمارين تجعلني أشعر بالراحة
11. التمارين تتيح لي الاتصال بأصدقائي والأشخاص الذين استمتع معهم
12. أنا أشعر بحرج شديد لممارسة التمارين
13. التمارين سوف تجنبني ارتفاع ضغط الدم
14. تكلفة ممارسة التمارين عالية جداً
15. التمارين تزيد من مستواي في اللياقة البدنية
16. أماكن التدريب ليس لديها جداول مريحة بالنسبة لي
17. عضلاتي تحسنت مع التمارين
18. التمارين تحسن من أداء نظام القلب والأوعية الدموية
19. أنا مرهق من ممارسة التمارين
20. مشاعر الرفاهية تحسنت لدى نتيجة ممارسة التمارين
21. زوجتي (أو شخص أخر مهم) لا يشجعني على ممارسة التمارين
22. ممارسة التمارين تزيد من قدرتي على التحمل
23. التمارين تحسن من مرونتي
24. التمارين تأخذ الكثير من وقت العلاقات الأسرية
25. تصرفي تحسن مع ممارسة التمارين

26. التمارين تساعدني على النوم بشكل أفضل ليلا
27. سوف أعيش لفترة أطول إذا كنت أماس التمارين
28. أعتقـد أن الناس في ملابس التمارين يبدون في صورة مضحكة
29. التمارين تساعدني على تقليل الشعور بالتعب
30. التمارين هي وسيلة جيدة بالنسبة لي لمقابلة أشخاص جدد
31. تحملي البدني تحسن من خلال ممارسة التمارين
32. التمارين تحسن من مفهوم الذات الخاص بي
33. أفراد عائلتي لا يشجعوني على ممارسة الرياضة
34. التمارين تزيد من يقظتي العقلية
35. التمارين تتيح لي الفرصة لتنفيذ الأنشطة العادية دون الشعور بالتعب
36. التمارين تحسن جودة عملي
37. التمارين تأخذ الكثير من الوقت الخاص بمسئوليات عائلتي
38. التمرين هو ترفيه جيد بالنسبة لي
39. التمارين تزيد قبولي من قبل الآخرين
40. التمارين بمثابة عمل شاق بالنسبة لي
41. التمارين تحسن الوظائف الجسدية بالنسبة لي
42. هناك عدد قليل من الأماكن لممارسة التمارين
43. التمارين تحسن من مظهر جسدي
