# Supplementary material for: Exercise Perceptions, Barriers, and Self-Efficacy Among Adults in Kuwait During the COVID-19 Pandemic
Source: Int J Environ Res Public Health. 2026 Apr 4;23(4):462. doi: 10.3390/ijerph23040462 (PMC13116150; doi:10.3390/ijerph23040462)
Supplement: Supplementary file 1 [file ijerph-23-00462-s001.zip › s2.pdf]

Supplementary Material. Tables S3 and S4 present the 13-item Barriers Specific Self-Efficacy Scale (BARSE) in English and Arabic. This instrument evaluates an individual's perceived capability (self-efficacy) to maintain an exercise routine (defined as exercising 3 times per week for the next 3 months) when faced with common social, personal, and environmental barriers.

Respondents rate their level of confidence for each hypothetical barrier on an 11-point percentage scale ranging from 0% to 100%, in 10-point increments. The scale is anchored by 0% ("Not at all Confident"), 50% ("Moderately Confident"), and 100% ("Highly Confident").

Table S3 - Barriers Specific Self-Efficacy Scale (BARSE) (English Version)

The following items reflect situations that are listed as common reasons for preventing individuals from participating in exercise sessions or, in some cases, dropping out. Using the scales below please indicate how confident you are that you could exercise in the event that any of the following circumstances were to occur.

Please indicate the degree to which you are confident that you could exercise in the event that any of the following circumstances were to occur by circling the appropriate %. Select the response that most closely matches your own, remembering that there are no right or wrong answers.

FOR EXAMPLE:
In question #1 if you have complete confidence that you could exercise even if “the weather was very bad,” you would circle 100%. If however, you had no confidence at all that you could exercise (that is, confidence you would not exercise), you would circle 0%.

0	10	20	30	40	50	60	70	80	90	100
Not at all Confident					Moderately Confident					Highly Confident

I believe that I could exercise 3 times per week for the next 3 months if:	**Not at all Confident**					**Moderately Confident**					**extremely confident**
1. The weather was very bad (hot, humid, rainy, cold).	**0**	**10**	**20**	**30**	**40**	**50**	**60**	**70**	**80**	**90**	**100**
2. I was bored by the program or activity.	**0**	**10**	**20**	**30**	**40**	**50**	**60**	**70**	**80**	**90**	**100**
3. I was on vacation.	**0**	**10**	**20**	**30**	**40**	**50**	**60**	**70**	**80**	**90**	**100**
4. I was not interested in the activity.	**0**	**10**	**20**	**30**	**40**	**50**	**60**	**70**	**80**	**90**	**100**
5. I felt pain or discomfort when exercising.	**0**	**10**	**20**	**30**	**40**	**50**	**60**	**70**	**80**	**90**	**100**
6. I had to exercise alone.	**0**	**10**	**20**	**30**	**40**	**50**	**60**	**70**	**80**	**90**	**100**
7. It was not fun or enjoyable.	**0**	**10**	**20**	**30**	**40**	**50**	**60**	**70**	**80**	**90**	**100**
8. It became difficult to get to the exercise location.	**0**	**10**	**20**	**30**	**40**	**50**	**60**	**70**	**80**	**90**	**100**
9. I didn't like the particular activity program that	**0**	**10**	**20**	**30**	**40**	**50**	**60**	**70**	**80**	**90**	**100**

I was involved in.											
10. My schedule conflicted with my exercise session.	0	10	20	30	40	50	60	70	80	90	100
11. I felt self-conscious about my appearance when I exercised.	0	10	20	30	40	50	60	70	80	90	100
12. An instructor does not offer me any encouragement.	0	10	20	30	40	50	60	70	80	90	100
13. I was under personal stress of some kind.	0	10	20	30	40	50	60	70	80	90	100

Table S4 - Barriers Specific Self-Efficacy Scale (BARSE) (Arabic Version)

تعكس العناصر التالية المواقف المدرجة كأسباب شائعة لمنع الأفراد من المشاركة في جلسات التمرين أو تركها في بعض الحالات.

باستخدام المقاييس أدناه، يرجى توضيح مدى ثقتك في أنه يمكنك ممارسة الرياضة في حالة حدوث أي من الحالات التالية
يرجى تحديد الدرجة التي تثق فيها أنه يمكنك ممارسة الرياضة في حالة حدوث أي من الحالات التالية من خلال وضع دائرة حول النسبة المئوية المناسبة. حدد الرد الذي يتطابق بشكل كبير مع إجابتك، وتذكر أنه لا توجد إجابات صحيحة أو خاطئة.

مثال على ذلك:

في السؤال رقم 1 إذا كنت واثقًا تمامًا من قدرتك على ممارسة الرياضة حتى لو "كان الطقس سيئًا للغاية"، فستحاط بدائرة بنسبة 100٪. ومع ذلك، إذا لم تكن لديك ثقة على الإطلاق في أنه يمكنك ممارسة الرياضة (أي الثقة التي لن تمارسها)، فستضع دائرة حول 0٪.

0	10	20	30	40	50	60	70	80	90	100
غير واثق على الاطلاق					واثق بدرجة متوسطة					واثق تماماً

	غير واثق على الاطلاق				واثق بدرجة متوسطة					واثق تماماً
١ .كان الطقس سيئًا جدًا (حار، رطب، ممطر، بارد).	0	10	20	30	40	50	60	70	80	90	100
٢ .شعرت بالملل من البرنامج أو النشاط الرياضي.	0	10	20	30	40	50	60	70	80	90	100
٣ .كنت في إجازة.	0	10	20	30	40	50	60	70	80	90	100
٤ .لم أكن مهتماً بهذا النشاط.	0	10	20	30	40	50	60	70	80	90	100
٥ .شعرت بالألم أو الانزعاج أثناء ممارسة الرياضة.	0	10	20	30	40	50	60	70	80	90	100
٦ .كان عليّ ممارسة الرياضة بمفردي.	0	10	20	30	40	50	60	70	80	90	100
٧ .إذا لم□كن الرياضة مرحة أو ممتعة.	0	10	20	30	40	50	60	70	80	90	100
٨ .أصبح من الصعب الوصو□ إلى مكان التمرين.	0	10	20	30	40	50	60	70	80	90	100
٩ .لم يعجبني برنامج النشاط المحدد الذي أشارك فيه.	0	10	20	30	40	50	60	70	80	90	100
١٠ .□عارض جدولي وار□باط□ي مع وقت جلسة التمرين.	0	10	20	30	40	50	60	70	80	90	100
١١ .شعرت بالخجل أو الحرج من مظهري أثناء ممارسة الرياضة.	0	10	20	30	40	50	60	70	80	90	100
١٢ .لم يقد□ لي المدرب أي□شجيع.	0	10	20	30	40	50	60	70	80	90	100
١٣ .كنت□حت ضغط نفسي أو□و□ر	0	10	20	30	40	50	60	70	80	90	100

											شخصي من أي نوع.
